# Ezrin Interacts with the SARS Coronavirus Spike Protein and Restrains Infection at the Entry Stage

**DOI:** 10.1371/journal.pone.0049566

**Published:** 2012-11-21

**Authors:** Jean Kaoru Millet, François Kien, Chung-Yan Cheung, Yu-Lam Siu, Wing-Lim Chan, Huiying Li, Hiu-Lan Leung, Martial Jaume, Roberto Bruzzone, Joseph S. Malik Peiris, Ralf Marius Altmeyer, Béatrice Nal

**Affiliations:** 1 HKU-Pasteur Research Centre, School of Public Health, The University of Hong Kong, Hong Kong SAR, China; 2 Department of Anatomy, School of Public Health, The University of Hong Kong, Hong Kong SAR, China; 3 Department of Pathology, School of Public Health, The University of Hong Kong, Hong Kong SAR, China; 4 Centre of Influenza Research, School of Public Health, The University of Hong Kong, Hong Kong SAR, China; 5 Department of Cell Biology and Infection, Institut Pasteur, Paris, France; 6 Institut Pasteur of Shanghai, Chinese Academy of Sciences, Shanghai, China; German Primate Center, Germany

## Abstract

**Background:**

Entry of Severe Acute Respiratory Syndrome coronavirus (SARS-CoV) and its envelope fusion with host cell membrane are controlled by a series of complex molecular mechanisms, largely dependent on the viral envelope glycoprotein Spike (S). There are still many unknowns on the implication of cellular factors that regulate the entry process.

**Methodology/Principal Findings:**

We performed a yeast two-hybrid screen using as bait the carboxy-terminal endodomain of S, which faces the cytosol during and after opening of the fusion pore at early stages of the virus life cycle. Here we show that the ezrin membrane-actin linker interacts with S endodomain through the F1 lobe of its FERM domain and that both the eight carboxy-terminal amino-acids and a membrane-proximal cysteine cluster of S endodomain are important for this interaction *in vitro*. Interestingly, we found that ezrin is present at the site of entry of S-pseudotyped lentiviral particles in Vero E6 cells. Targeting ezrin function by small interfering RNA increased S-mediated entry of pseudotyped particles in epithelial cells. Furthermore, deletion of the eight carboxy-terminal amino acids of S enhanced S-pseudotyped particles infection. Expression of the ezrin dominant negative FERM domain enhanced cell susceptibility to infection by SARS-CoV and S-pseudotyped particles and potentiated S-dependent membrane fusion.

**Conclusions/Significance:**

Ezrin interacts with SARS-CoV S endodomain and limits virus entry and fusion. Our data present a novel mechanism involving a cellular factor in the regulation of S-dependent early events of infection.

## Introduction

Coronaviruses (CoV) are enveloped, single strand positive-sense RNA viruses capable of infecting a wide range of birds and mammals, including humans [1]–[5]. In 2003, the causative agent of the severe acute respiratory syndrome (SARS) outbreak was identified to be a novel, highly pathogenic, respiratory human coronavirus, the SARS-CoV [6]–[9].

The viral envelope glycoprotein Spike (S) is a highly glycosylated trimeric type I membrane protein, which forms the typical spike structures at the envelope of coronaviruses. S is responsible for binding to cellular receptors and envelope fusion with host cell membranes. SARS-CoV S is 1255 amino-acids long. Although the exact boundaries of S transmembrane domain are not clearly defined, the N-terminal ectodomain and the C-terminal endodomain are most likely 1193 and 39 amino-acids long, respectively.

SARS-CoV S mediates binding to the virus' cellular receptor, the angiotensin-converting enzyme 2 (ACE-2) on apical surfaces of epithelial cells [10], [11]. S cleavage into S1 and S2 subunits by either extracellular proteases [12] or the pH-sensitive endosomal protease cathepsin-L [13] is a prerequisite for its fusogenicity. This cleavage triggers the release of a hydrophobic fusion peptide at the N-terminal end of the S2 subunit, which inserts itself into host membranes, placing viral and cellular membranes at close proximity. S conformational changes lead to the fusion of the viral envelope with cellular membranes. Membrane destabilization by the S juxtamembrane domain, localized in the ectodomain, as well as clusters of palmitoylated cysteines, found within the membrane-proximal half of the S endodomain, may facilitate the fusion process [14], [15].

A recent study has shown that a palmitoylation-null mutant S protein is deficient for partitioning into detergent resistant membranes and for mediating S-dependent cell-cell fusion [16]. Other regions within the endodomain may also regulate fusion and S-mediated entry into susceptible cells [17]–[19]. A C-terminal truncation of 17 residues was shown to increase S-mediated cell-to-cell fusion [17]; a truncation of the last 19 amino acids of S resulted in higher levels of transduction of Vero cells by murine leukemia virus (MLV) S-pseudotyped particles [19]. However it is unclear whether cellular factors bind to S endodomain and regulate fusogenicity.

S endodomains are accessible to cellular machineries i/ during production, maturation, trafficking, and assembly of viral envelope proteins (pre-budding steps), and ii/ at a critical stage of virus entry, just after formation of the fusion pore, and after fusion. We hypothesized that during these crucial stages of infection, interactions between the S endodomain and cellular machineries could participate in regulating infectivity and host cell susceptibility to infection.

We performed a yeast two-hybrid screening using S endodomain as bait. Here we describe for the first time the interaction between SARS-CoV S endodomain and ezrin, a member of the Ezrin/Radixin/Moesin (ERM) family of proteins. ERM have a crucial role in organizing membrane domains through their ability to interact with lipid rafts, transmembrane proteins and filamentous actin [20]. Hence, they mediate a fine-tuned linkage that strengthen the cell cortex, partition membrane clusters, and regulate signal transduction pathways [20]. Recent studies suggest that ezrin participates in the formation of either a diffusion barrier or a tether, which limits mobility of membrane components, in particular lipid rafts [21], [22].

Ezrin is found in a cytosolic dormant state where the C-terminal ERM-association domain, the C-ERMAD, and the N-terminal FERM domains interact with each other, quenching protein binding sites, and is activated through phosphatidyl-inositol 4,5 biphosphate binding and phosphorylation [20], [23]. Activation opens up the molecule and releases the FERM and C-ERMAD domains, which are then free to interact with membranes (phospholipids and endodomains of transmembrane proteins) and signaling molecules, and actin cytoskeleton, respectively [20], [24], [25]. Overexpression of the FERM domain of ezrin has a dominant negative effect, by saturating ezrin binding sites at the plasma membrane and inhibiting its interaction with actin by blocking C-ERMAD sites [20], [26].

Interestingly, viruses such as vaccinia virus and minute virus of mice were shown to modulate the pathways in which ERM proteins are involved to ensure viral entry and cell spreading, respectively [27], [28]. Furthermore, ERM proteins have been shown to have differential regulating roles in retroviral life cycles, depending on the viral step examined [29]–[32]. Therefore, ERM proteins, which are crucial regulators of cell functions, may play important roles at various stages of virus life cycles.

Our objectives were to confirm the interaction between SARS-CoV S and ezrin and to study the role of ezrin in S-dependent entry events. Here we show a novel restraining role of ezrin at early stages of SARS-CoV replication cycle. We discuss the relevance of our findings in the regulation of SARS-CoV entry in host cells.

## Results

### The endodomain of the SARS-CoV S protein binds to the F1 lobe of the FERM domain of ezrin in yeast

**Figure 1:**
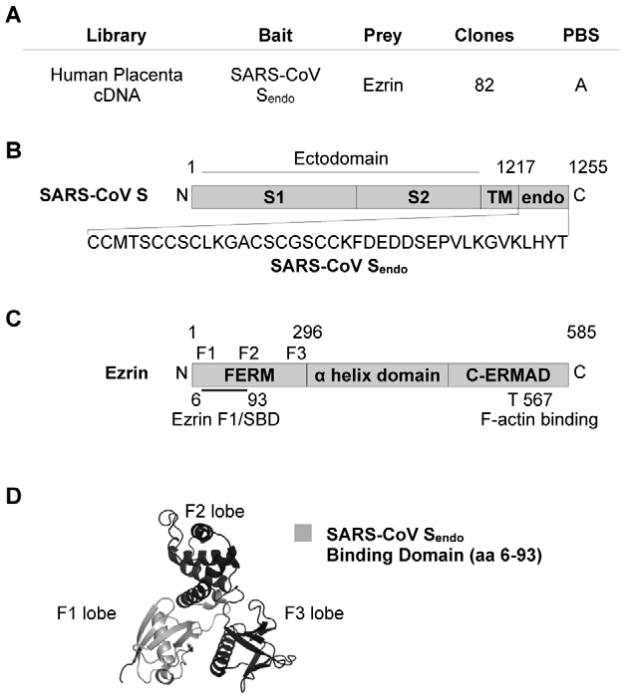
The F1 lobe of the ezrin FERM domain binds to the SARS-CoV S endodomain. A. Table summarizing the yeast two-hybrid screening results for ezrin - S endodomain interaction. B. Schematic representation of the SARS-CoV S endodomain sequence (not drawn to scale). S1: Subunit 1. S2: Subunit 2. TM: transmembrane domain. endo: endodomain. C. Schematic representation of ezrin (not drawn to scale). The bold line represents the common sequence of the 82 cDNAs corresponding to ezrin found in the yeast two-hybrid screening. The domain was named ezrin F1/Spike Binding Domain (SBD). F1, F2, and F3 represent approximate regions corresponding to the three lobes of the FERM domain. T567: threonine 567. D. Representation of the three-dimensional crystal structure of the ezrin FERM domain, showing the three distinct globular lobes F1, F2, and F3. The region in light grey represents amino-acids of the F1/SBD. The FERM domain crystal structure for ezrin (PDB ID: 1NI2) was downloaded from the Protein Data Bank (http://www.pdb.org). 3-D rendering of the ezrin FERM domain was performed using MacPyMol software (DeLano Scientific).

We hypothesized that cellular proteins may interact with the carboxy-terminal endodomain of the S envelope protein and either help or restrict processes involving S. To identify such cellular factors, we performed a genomic yeast two-hybrid screen using the SARS-CoV S endodomain as bait (Fig. 1A–B). A random-primed cDNA library from human placenta was screened and 233 positive clones were identified. The most prominent result of the screen was the identification of the interaction between S endodomain and the cellular protein ezrin (Fig. 1A, C) with a total of 82 positive clones (35%) corresponding to this protein (Accession number: NM_003379; Fig. 1C). The S - ezrin interaction was classified with high confidence score (Predicted Biological Score = A; Fig. 1A) and was not found in parallel screens performed with other viral baits [33], [34] and data not shown). These data indicate that ezrin specifically interacts with SARS-CoV S endodomain in yeast.

To determine the binding region of ezrin to S endodomain, the 82 ezrin cDNA sequences were aligned using ClustalW multiple sequence alignment program. All ezrin cDNA shared a common sequence of 264 base pairs corresponding to amino acids 6 to 93 (Fig. 1C). Interestingly, this region corresponds almost completely to the F1 lobe of the N-terminal FERM domain (amino acids 2 to 82 [35], Fig. 1C and 1D). This suggests that interaction of ezrin with S endodomain requires a complete F1 lobe. This region was subsequently named ezrin F1/S Binding Domain or F1/SBD (Fig. 1C).

### S endodomain is sufficient to pull-down ezrin from epithelial cell lysates

**Figure 2:**
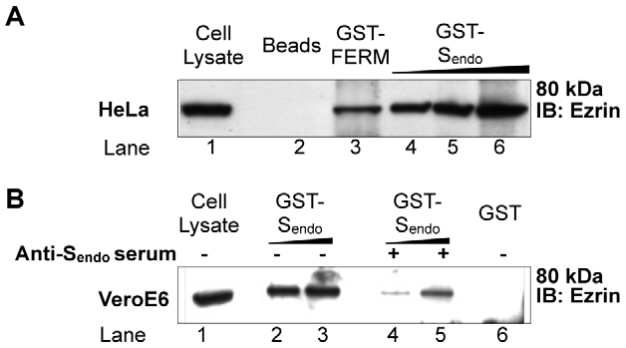
*In vitro* confirmation of the interaction between SARS-CoV S endodomain and ezrin. A. The endodomain of S pulls down ezrin from HeLa cell lysate. Cell lysate was incubated with Glutathione-Sepharose beads either uncoupled (lane 2) or coupled with the fusion protein GST-FERM (positive control, lane 3) or increasing amounts of GST-S_endo_ (lanes 4–6). B. The endodomain of S pulls down ezrin from Vero E6 cell lysate. Similarly, cell lysate was incubated with Glutathione-Sepharose beads bound either to GST fused to S_endo_ in absence (lanes 2–3) or presence (lanes 4–5) of rabbit serum that recognizes the endodomain of S, or to GST alone (lane 6). For both A. and B. 10 µL of cell lysate was used as input control which represents 1.6% and 4% of the volume used for the pull down for A. and B., respectively. IB: Immunoblot. Results shown are representative of two independent experiments.

To confirm the interaction of S endodomain with human ezrin, GST-pull down assays were performed. Briefly, we used GST-fused S endodomain (GST-S_endo_) protein bound to glutathione-coupled sepharose beads to pull down endogenous ezrin protein from cell lysates of HeLa human epithelial cells (Fig. 2A). Glutathione-sepharose beads alone and GST protein fused to the ezrin FERM domain and bound to beads (GST-FERM) were used as negative and positive controls, respectively (Fig. 2A lanes 2–3). GST-FERM was able to pull down ezrin from the HeLa lysate. This result was expected as the FERM domain has the property to specifically interact with the C-ERMAD of ezrin. Furthermore, GST-S_endo_ could coprecipitate ezrin in a dose-dependent manner (Fig. 2A lanes 4, 5, 6). This result confirms the interaction of the SARS-CoV S endodomain with the human ezrin protein.

We then decided to analyze the interaction of the S endodomain with ezrin from the SARS-CoV-permissive Vero E6 cell line. Similarly, the GST-S_endo_ could precipitate the Vero E6 endogenous ezrin protein (Fig. 2B lanes 2–3). Incubation of GST-S_endo_ with Vero E6 cell lysates in the presence of rabbit serum against the endodomain of S significantly diminished the interaction (Fig. 2B lanes 4–5). Moreover, GST protein alone was not able to pull down ezrin (Fig. 2B lane 6), further confirming that the 39 amino-acids of SARS-CoV S endodomain are responsible for the interaction. These results collectively show that the S endodomain is capable of interacting specifically with ezrin *in vitro*.

### The last 8 carboxy-terminal residues and the membrane proximal cysteine cluster of SARS-CoV S endodomain are involved in ezrin binding in vitro

**Figure 3:**
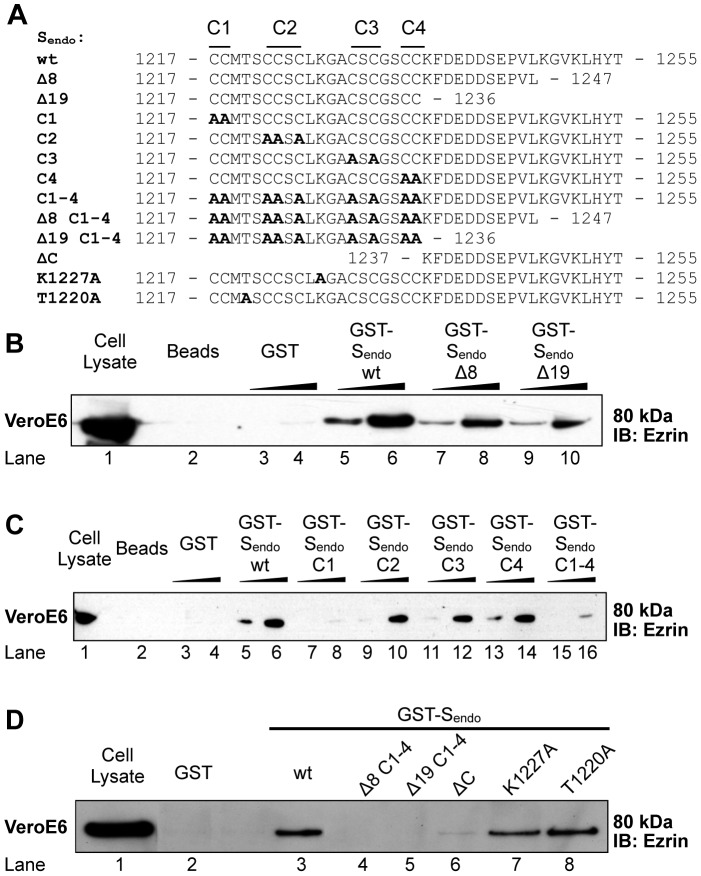
Characterization of interactions determinants of S endodomain binding to ezrin. A. Sequences of the wild type (wt) and mutated S endodomain used in the GST-pull down analysis. Twelve GST fusion proteins were produced with mutations in the S endodomain. Δ indicates truncations and C indicates cysteine to alanine mutations (in bold) of cysteine clusters (1 to 4). B. & C. Effect of truncations or cysteine to alanine mutations. Vero E6 lysate was incubated with Glutathione-Sepharose beads coupled or not with GST, GST-S_endo_ _wt_ (B. & C.), GST-S_endo Δ8_, and GST-S_endo Δ19_(B.), GST-S_endo C1_, GST-S_endo C2_, GST-S_endo C3_, GST-S_endo C4_, and GST-S_endo C1-4_ (C.) using 1 or 5 µg of GST fusion proteins. D. Effects of cysteine to alanine mutations and truncations or point mutations (K1227A and T1220A). Vero E6 lysate was incubated with Glutathione-Sepharose beads coupled with either GST, GST-S_endo wt_, GST-S_endo__Δ8 C1-4_, and GST-S_endo__Δ19 C1-4_, GST-S_endo_ _ΔC_, GST-S_endo K1227A_, GST-S_endo__T1220A_ using 1 µg of each GST fusion protein. 5 µL of cell lysate was used as input control for B. & C. (8% of volume used in each pull down); 10 µL of cell lysate was used as input control for D. (5% of volume used in each pull down). IB: Immunoblot. Results shown are representative of two independent experiments.

To map the amino-acids on S that are important for the interaction with ezrin, we decided to construct a series of S endodomain mutants and analyze their capacity to bind ezrin in GST pull down assays (Fig. 3A). Analysis of the SARS-CoV S endodomain sequence revealed three regions with distinctive characteristics: a carboxy-terminal basic region (amino-acids 1248–1254), a central acidic stretch (amino-acids 1239–1244) and a series of clusters of cysteine residues (amino-acids 1217–1236). We reasoned that these regions could be involved in the interaction with ezrin. First, we constructed truncation mutants by deleting the carboxy-terminal regions either containing the basic cluster or both the basic cluster and the acidic stretch. These truncation mutants consisted in deletion of eight and nineteen amino-acids, respectively (S_endo Δ8_ and S_endo Δ19_, Fig. 3A). As expected, glutathione-conjugated beads and GST-coupled beads could not pull down ezrin whereas beads coupled to GST-S_endo wt_ could precipitate ezrin efficiently from a Vero E6 cell lysate (Fig. 3B lanes 2–6). Both Δ8 and Δ19 truncations were able to significantly diminish interaction with ezrin at similar levels, but not abrogate it (Fig. 3B lanes 7, 8, 9, 10). These data show that the eight carboxy-terminal amino-acid stretch, which is rich in basic residues, is important for ezrin binding and that another motif in the endodomain may contribute to the interaction. Conversely, the acidic stretch does not seem involved in the interaction.

We then analyzed the implication of the nine cysteines in the interaction with ezrin. These residues are found in the membrane proximal region of the S endodomain. To simplify the analysis, we decided to pool cysteines into four clusters, namely C1 to C4, of two to three residues (Fig. 3A). While mutations of C2, C3, and C4 clusters had little impact on the interaction with ezrin (Fig. 3C compare lanes 9–14 with lanes 5–6), mutation of the cluster C1 was able to almost completely abrogate the interaction (Fig. 3C lanes 7–8). Consistently, mutation of all clusters of cysteines also greatly reduced binding to ezrin (Fig. 3C lanes 15–16). This result indicates that the C1 membrane proximal cysteine cluster is important for binding to ezrin *in vitro*.

Since endodomain truncations and cysteine to alanine mutations of the SARS-CoV S endodomain were found to diminish interaction with ezrin individually, both types of modifications were next tested simultaneously (S_endo Δ8__C1-4_ and S_endo Δ19 C1-4_). Additionally, a truncation mutant was tested where the cysteine cluster-containing portion (residues 1217 to 1236) of S endodomain was deleted (S_endo ΔC_). Also, the positively-charged residue lysine 1227 and the polar residue threonine 1220 were point-mutated into alanines to investigate their possible involvement in the S-ezrin interaction (S_endo K1227A_ and S_endo T1220A_; Fig. 3A). As expected, the ΔC truncation almost completely abrogated the interaction with ezrin, although a faint band was still observed further indicating that the C-terminal half of S endodomain contains some interaction determinants (Fig. 3D lane 6). The point mutation on the lysine K1227 did not significantly affect binding with ezrin. The point mutation on the threonine T1220 had no effect on binding with ezrin. Confirming our previous results, S endodomain mutants containing both truncations (Δ8 or Δ19), and the cysteine mutations (C1-4) were not able to bind ezrin (Fig. 3D lanes 4–5). Together, these data show that the membrane proximal cysteine cluster C1 and the last eight C-terminal residues are important determinants of interaction of SARS-CoV S endodomain with ezrin *in vitro*.

### Ezrin is present at the site of entry of Spike-pseudotyped lentiviral particle

**Figure 4:**
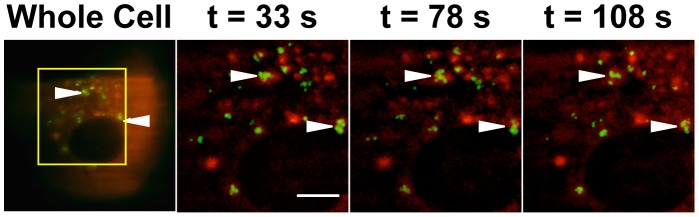
Ezrin accumulates at sites of entry of SARS-CoV S pseudotyped particles. Vero E6 cells stably expressing RFP-ezrin were inoculated with SARS-CoV S-pseudotyped lentiviral particles harboring a GFP-tagged Vpr protein (SARSpp GFP-Vpr) on ice for 30 minutes. Unbound particles were washed. Internalization of particles was induced by placing the culture dish in a 37°C 5% CO_2_ chamber. At 30 minutes post temperature switch (t = 0), cells were analyzed by TIRFM. Time-lapse images were acquired every 3 seconds. The whole cell (first frame) is shown on the left panel. The region shown for time-lapse images is indicated by a square. 3 frames at t = 33, 78 and 108 seconds out of a total of 50 frames are shown and correspond to the times after the start of image acquisitions. The movie of this image sequence is shown as supporting material (Supporting movie S1). Scale bar = 40 µm.

Considering the major role of S in virus entry and membrane fusion, we first assessed whether S endodomains could be accessible to ezrin at early stages of infection. To mimic SARS-CoV entry, we used Spike-pseudotyped lentiviral particles (SARSpp), which were previously shown to enter cells in an ACE-2, cathepsin-L, and low pH dependent pathway, faithfully recapitulating the entry process of native SARS-CoV virions [36]–[39]. Vero E6 epithelial cells stably expressing RFP-ezrin fusion protein were infected with GFP-Vpr SARSpp. At 30 minutes post-infection, cells were analyzed using a total internal reflection fluorescence microscope (TIRFM) to visualize events occurring at proximity of the plasma membrane and minimize background from the other layers of the cells. Green dots corresponding to GFP-Vpr SARSpp were readily observed and found associated with RFP-ezrin enriched domains (Fig. 4, white arrowheads, Supporting movie S1). This result indicates that ezrin is present at sites of SARSpp entry, possibly coating endosomes following internalization of particles. Similar data were obtained using a spinning disc confocal microscope on live cells expressing RFP-ezrin (data not shown). This would suggest that after the formation of the fusion pore, S endodomains facing the host cell cytosol have the possibility to interact with ezrin molecules.

### Knock down of ezrin by siRNA increases SARS-CoV S-mediated entry

**Figure 5:**
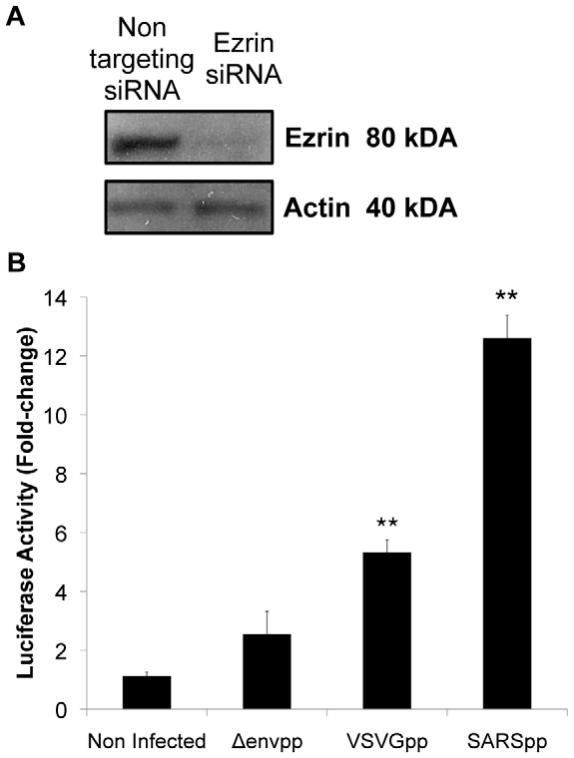
Knock down of ezrin by siRNA increases entry of SARS-CoV S pseudotyped particles. A. Ezrin expression knock down by siRNA. HeLa-F5 cells stably expressing the SARS-CoV receptor ACE2 were transfected twice with ezrin or non-targeting siRNAs and knock down efficiency was estimated by Western blot analysis of ezrin and actin content of cell lysates. B. Luciferase activity fold change analysis. HeLa-F5 cells treated with siRNAs as indicated above were infected with pseudotyped lentiviral particles harboring the VSV G or SARS-CoV S viral envelope glycoproteins. Δenvpp indicates lentiviral particles without any viral surface glycoprotein. The fold change in luciferase activity was calculated using the non-targeting siRNA condition as negative control. Experiments were performed in triplicates and the results of this experiment are representative of at least three independent experiments. ** indicates a value of *p*<0.001 in two-tailed t-tests.

To investigate the potential role of ezrin in S-dependent entry, we decided to silence the expression of ezrin by specific siRNA. HeLa-F5 cells, stably expressing the SARS-CoV receptor ACE2, were transfected with siRNAs that target ezrin mRNA or non-targeting control siRNAs. The ezrin siRNA transfection decreased expression significantly, with a quantified 80% knock down at the protein level (Fig. 5A). Ezrin siRNA was shown to slightly increase transduction levels by Δenvpp and VSVGpp (around 3- and 5-fold increases, respectively) (Fig. 5B). Interestingly, the highest fold-change observed was for SARSpp where S-mediated entry was enhanced more than 12-fold. These data indicate that ezrin expression silencing has a slight enhancing effect on entry of viral particles uptake in general, as well as a more specific highly increasing effect on entry of SARS-CoV S pseudotyped particles. This suggests a negative regulatory role for ezrin in SARS-CoV S-mediated entry.

### Mutation of the ezrin-binding domain on S endodomain favors SARSpp entry into Vero E6 cell

**Figure 6:**
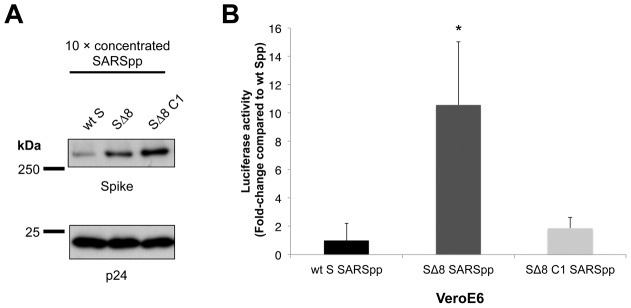
S C-terminal mutations that decrease ezrin interaction enhance transduction by pseudotyped particles in Vero E6 cells. A. Generation of lentiviral pseudotyped particles harboring wild-type (wt) or mutated (Δ8 and Δ8 C1) SARS-CoV Spike proteins. A Western blot assay was performed on concentrated SARSpp particles where the Spike protein and the lentiviral backbone protein p24 were probed. Estimation of protein quantities was performed using densitometry analysis. B. Entry of wt and mutated SARS-CoV S pseudotyped particles. Vero E6 cells were infected by wt S SARS-CoV pseudotyped particles, along with mutated SARSpp (SΔ8 SARSpp and SΔ8 C1 SARSpp). Results are expressed as fold-change in luciferase activity compared to the wt S SARSpp. The results are averages of triplicates and are representative of at least three independent experiments. * indicates a value of *p*<0.05 in a two-tailed t-test.

To confirm that ezrin binding to S endodomain negatively regulates SARSpp entry, we have generated mutated SARS-CoV S proteins that contain modified endodomains and pseudotyped them. The mutated SARS S pseudotyped particles were designed by taking into account the biochemical pull-down data we have established (Fig. 3). To completely abolish ezrin interaction, we constructed a S mutant with cysteine to alanine mutations of the first cysteine cluster and with a deletion of the last 8 amino acids (SΔ8 C1). Considering that the C1 mutation could have severe consequences on fusion [14], we also generated a second S mutant with only a deletion of the 8 last amino acids of the C-terminus (SΔ8), a modification which should partially alter interaction with ezrin. SΔ8 and SΔ8 C1 S proteins could be incorporated into lentiviral particles (Fig. 6A, upper panel) and similar amounts of particles were found in concentrated supernatants, as indicated by detection of the HIV p24 protein (Fig. 6A, lower panel). Levels of incorporation of wt S, SΔ8 and SΔ8 C1 were quantified by densitometry and normalized to p24 levels. The ratio of S incorporation was 1 ∶ 2.5 ∶ 3.9 for wt S, SΔ8 and SΔ8 C1, respectively. Entry of mutated S pseudotyped particles along with wt S SARSpp was analyzed in SARS-CoV-susceptible Vero E6 cells (Fig. 6B). Both mutated SARSpp were found to transduce Vero E6 cells more efficiently than wt S SARSpp. Although mutation of C1 is expected to induce a defect in fusion [14], a 2-fold increase in transduction was measured for SΔ8 C1 SARSpp. This increase can be explained by both the efficient incorporation of SΔ8 C1 in pseudotyped particles and the lack of interaction with ezrin. Interestingly, significantly higher levels of transduction were observed for the SΔ8 SARSpp, with an increase of transduction of approximately 11-fold compared to wt S SARSpp. This result is in agreement with previously published data where murine leukemia virus (MLV)-based SΔ8 SARSpp were found to transduce Vero cells more efficiently than wt S pseudotyped particles [19]. In our study, better incorporation of SΔ8 could be in part responsible for the increase of transduction. However, the difference in fold-change that was observed for SΔ8 incorporation into pseudoparticles and cellular transduction (2-fold compared to 11-fold increase, respectively) suggests that partial disruption of interaction with ezrin contributes to this enhancement of transduction. Taken together, these data are in agreement with the hypothesis that ezrin binding to S endodomain decreases entry of viral particles.

### Expression of the ezrin FERM domain increases susceptibility of Vero E6 cells to SARS-CoV infection

**Figure 7:**
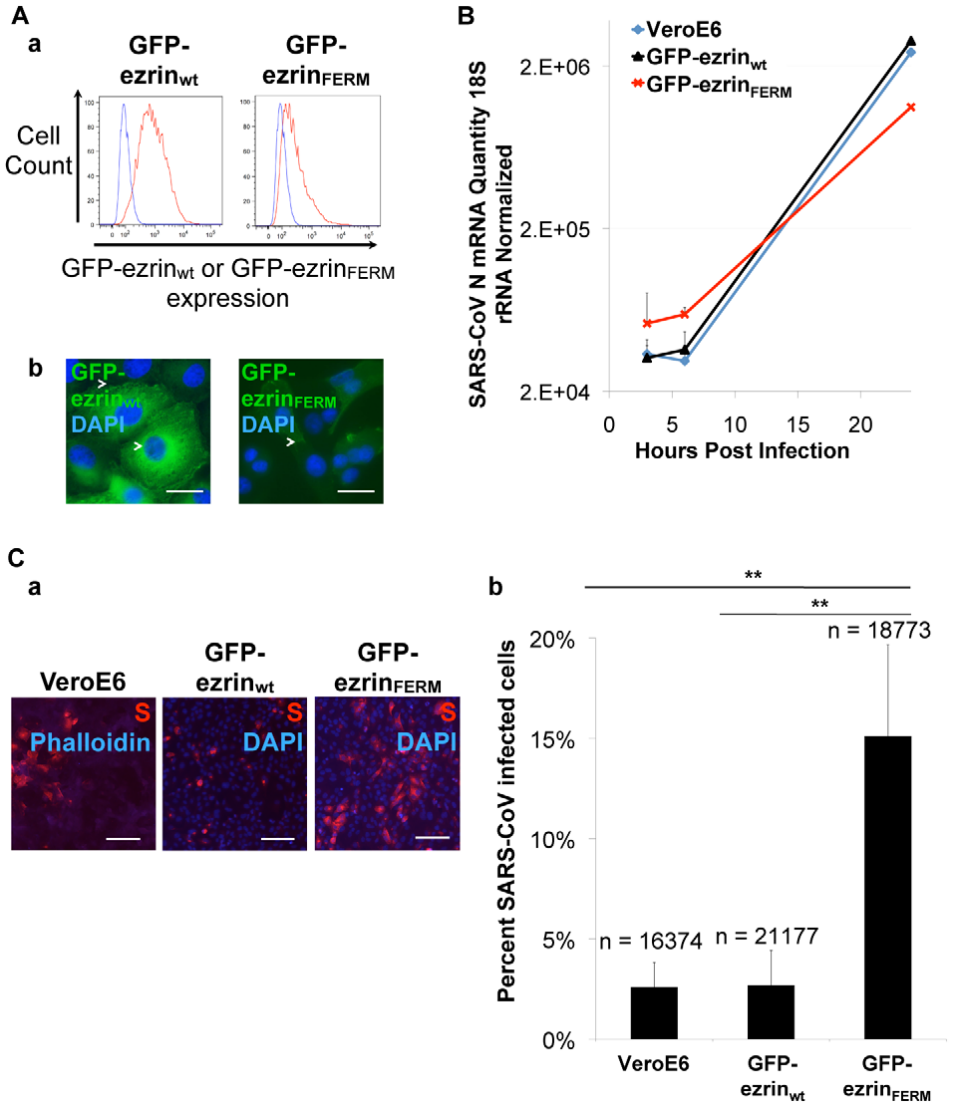
Expression of the FERM domain of ezrin increases Vero E6 cell susceptibility to SARS-CoV infection. A. Flow cytometry analysis (a.) and subcellular localization (b.) of the wt or FERM domain GFP-ezrin in clonal Vero E6 stable cell lines. (b.) Arrows indicate enrichments of wt or FERM ezrin. Scale bars: 20 µm. B. Time course of SARS-CoV replication in Vero E6 stable cell lines expressing wt or FERM GFP-ezrin. Vero E6, Vero E6 GFP-ezrin_wt_, Vero E6 GFP-ezrin_FERM_ were infected with SARS-CoV (strain HK39849) at M.O.I. 5. At 3, 6 and 24 hours post infection, SARS-CoV N RNA levels were measured using qRT-PCR with 18S rRNA normalization. For each condition the average of two measurements of two independent wells was calculated. C. SARS-CoV infection rates in Vero E6, Vero E6 GFP-ezrin_wt_ and Vero E6 GFP-ezrin_FERM_ cell lines. Cells were infected with SARS-CoV (strain HK39849) at M.O.I. of 5. 24 h post-infection, cells were immunolabeled for SARS-CoV S. (a.), scale bars: 100 µm. (b.) for each cell line, images of ten random microscopy fields were acquired and analyzed for total number of cells (n; DAPI or Phalloidin AMCA staining) and SARS-CoV S positive cells (TRITC staining) using Imaris 6.3 software. ** indicates a value of *p*<0.001 in two-tailed t-tests.

To further study the role of ezrin during SARS-CoV infection, we produced clonal Vero E6 stable cell lines that either express wild-type ezrin (ezrin_wt_) or the N-terminal FERM domain of ezrin (ezrin_FERM_) fused to the green fluorescent protein (GFP). The latter form of ezrin is known to have a dominant negative effect on endogenous ezrin [26]. The levels of expression and subcellular localization of GFP-ezrin_wt_ and GFP-ezrin_FERM_ in selected clones were monitored by flow cytometry and fluorescence microscopy, respectively (Fig. 7A a–b). Flow cytometry analysis showed that clones GFP-ezrin_wt_ and GFP-ezrin_FERM_ had means of fluorescence intensities of 2×10^3^ and 7×10^2^, respectively (Fig. 7A a). As expected, GFP-ezrin_wt_ distributed in the cell cytosol with occasional enrichments at the cell cortex, whereas GFP-ezrin_FERM_ was almost exclusively found at the cell cortex, in membrane ruffles and lamellipodia (Fig. 7A b). We verified that Vero E6, and clones GFP-ezrin_wt_ and GFP-ezrin_FERM_ expressed similar levels of ACE-2 receptor at cell surface (data not shown).

The stable cell lines and control Vero E6 cells were first infected with SARS-CoV. Infection levels were then monitored by quantitative real-time PCR (qRT-PCR) on the viral N gene at 3, 6 and 24 hours post infection (Fig. 7B) and by immunofluorescence assay at 24 hours post-infection (Fig. 7C). During the whole time course of infection, levels of N RNAs measured in infected GFP-ezrin_wt_ cells were comparable to those counted for control Vero E6 cells (Fig. 7B). Normalized N levels at early time points ranged between 3.2×10^4^ and 3.6×10^4^ and reached approximately 2.6×10^6^ at 24 hours post-infection for these two cell lines (∼2 log increase between 6 and 24 hours). Interestingly, although the replication rate at 24 hours was lower in clone GFP-ezrin_FERM_ than control Vero E6 cells (normalized N levels of 1×10^6^), a slight enhancement was observed at early time points (normalized N RNAs ranged between 5.2×10^4^ and 6×10^4^). Similar profiles were observed for qRT-PCR on viral ORF1b (data not shown). This result shows that expression of the dominant negative FERM domain of ezrin slightly enhances early, but not late stages of infection.

In parallel to qRT-PCR analysis, cells were analyzed by immunolabeling and fluorescence microscopy at 24 hours post-infection (Fig. 7C a). S was labeled to mark infected cells. All cells were stained for either actin or nuclei. For each cell line, the percentage of infected cells was calculated (Fig. 7C b). Infection rates in Vero E6 and Vero E6 GFP-ezrin_wt_ cells were similar with 2.5% of S-positive cells. Interestingly, a 5.8-fold increase in the percentage of infected cells (15% of cells) was observed for the GFP-ezrin_FERM_ cell line, when compared with control cells. This higher susceptibility to infection is consistent with higher levels of N RNAs detected at early time points post-infection for the clone GFP-ezrin_FERM_. These data indicate that partial disruption of ezrin function by expression of its dominant negative form increases host cell susceptibility to infection.

### Expression of the ezrin FERM domain increases S-mediated entry

**Figure 8:**
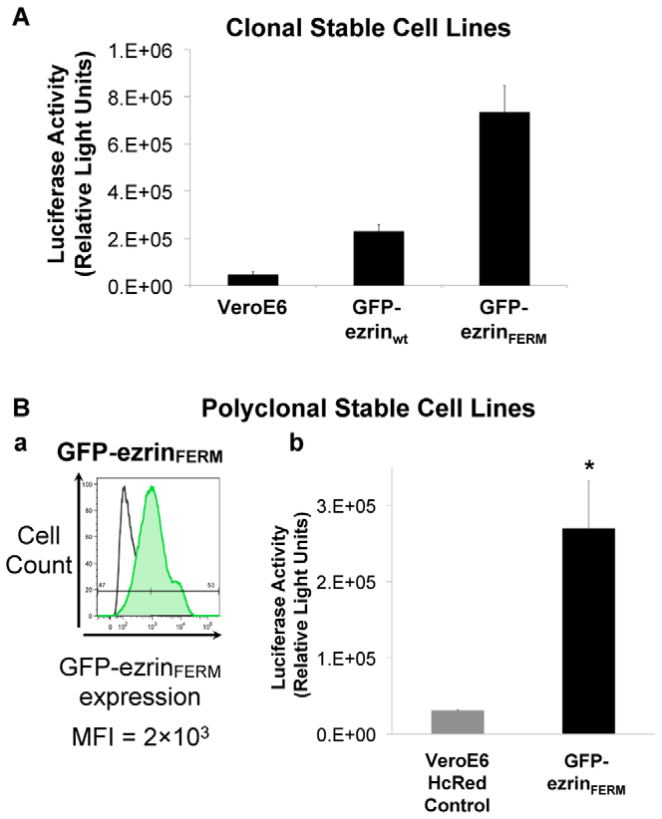
Expression of the FERM domain of ezrin enhances entry of SARS-CoV S pp. A. Vero E6, Vero E6 GFP-ezrin_wt_ and Vero E6 GFP-ezrin_FERM_ clonal cells were infected with SARSpp and incubated for 72 hours and the activity of luciferase was measured (results correspond to average of triplicates). B. (a.) Flow cytometry analysis of polyclonal Vero E6 stable cells expressing GFP-ezrin_FERM_. Lentiviral vector transductions enabled Vero E6 cells to express irrelevant HcRed or GFP-ezrin_FERM_ proteins. MFI: Mean Fluorescence Intensity. (b.) Polyclonal Vero E6 cells stably expressing HcRed (negative control) or GFP-ezrin_FERM_ were infected with SARSpp, incubated for 72 hours, and the activity of luciferase was measured. The results of this experiment are representative of at least three independent experiments. * indicates a value of *p*<0.05 in two-tailed t-tests.

To verify that the enhanced permissiveness to SARS-CoV infection observed in cells expressing the ezrin FERM domain is due to a higher efficiency of entry of virions, we investigated levels of transduction of control, GFP-ezrin_wt_ and GFP-ezrin_FERM_ clonal Vero E6 cells by SARSpp (Fig. 8A). Interestingly, whereas the transduction level of GFP-ezrin_wt_ was not significantly different than in control Vero E6 cells, GFP-ezrin_FERM_ cells showed a ∼15-fold increase in luciferase activity values. This result indicates that clone GFP-ezrin_FERM_ is more permissive to SARS-CoV S-mediated entry and is consistent with the higher rates of infection observed with SARS-CoV in this cell line (Fig. 7B–C).

To exclude a clonal effect, a new batch of polyclonal Vero E6 cells expressing GFP-ezrin_FERM_ was generated. As control, we used Vero E6 cells transduced to express a non-relevant HcRed protein. Expression level of GFP-ezrin_FERM_ was monitored by flow cytometry (Fig. 8B a). These cells were used to carry out infections using SARSpp (Fig. 8B b). Consistently with previous data on clonal cell lines, polyclonal cells expressing GFP-ezrin_FERM_ showed a 9-fold increase in luciferase activity, indicating higher susceptibility to SARSpp infection. Of note, SARSpp infection was not enhanced in cells selected to express high amounts of GFP-ezrin_FERM_ (data not shown). Altogether, our data indicate that partial disruption of ezrin activity by expression of low levels of GFP-ezrin_FERM_ enhances cell susceptibility to S-mediated entry and support a restraining role of ezrin at this stage.

### Expression of the FERM domain of ezrin by target cells enhances S-mediated cell-cell fusion

**Figure 9:**
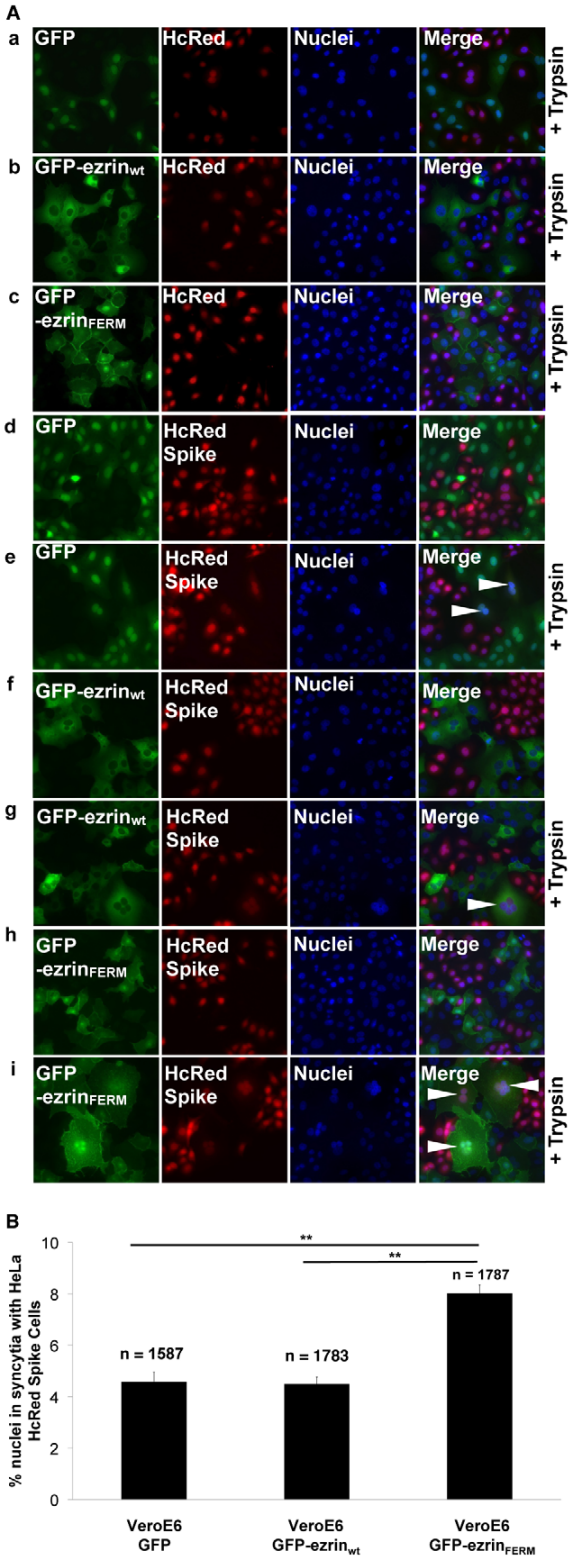
Effect of wt or FERM ezrin expression on S-mediated cell-cell fusion. A. Microscopy analysis of syncytia formation induced by activated SARS-CoV S. Vero E6 GFP, Vero E6 GFP-ezrin_wt_ and Vero E6 GFP-ezrin_FERM_ cells were overlaid on HeLa cells stably expressing HcRed alone or HcRed and SARS-CoV S. SARS-CoV S was activated or not with trypsin for 15 minutes (+Trypsin). 18 hours later, cells were fixed, nuclei stained with DAPI and analyzed by microscopy for syncytia. Arrows indicate syncytia. B. Quantification of syncytia. For the conditions in which HeLa HcRed Spike cells (+Trypsin) were incubated with Vero E6 GFP or Vero E6 GFP-ezrin_wt_ or Vero E6 GFP-ezrin_FERM_ cells, 10 random microscopy fields were analyzed for total number of nuclei (DAPI) and number of nuclei in multi-nucleated cells (DAPI/HcRed/GFP). Results are representative of three independent experiments. ** indicates a value of *p*<0.001 in two-tailed t-tests.

Our previous functional experiments have demonstrated a negative regulatory role for ezrin during S-mediated entry. To characterize further this phenomenon, we questioned whether ezrin could be involved in the S-mediated fusion process, during which S endodomains become accessible to the cytosol. To that end, we chose to study the effect of expression of the FERM domain of ezrin on S-dependent fusogenicity in an *in vitro* cell-cell fusion assay. In this experiment, HeLa cells stably expressing both S and a HcRed fluorescent marker (HeLa HcRed Spike) were co-incubated with GFP, GFP-ezrin_wt_ or GFP-ezrin_FERM_ Vero E6 stable cell lines. As expected, control HeLa HcRed cells were not able to fuse with any of the three Vero E6 cell lines, as no syncytia were found (Fig. 9A panels a to c). Similarly, no syncytia were observed for HeLa HcRed Spike cells incubated with Vero E6 stable cell lines but not activated by trypsin treatment (Fig. 9A panels d, f and h). Conversely, about 4.5% of nuclei were found in syncytia when HeLa HcRed Spike cells were incubated in presence of either GFP or GFP-ezrin_wt_ Vero E6 cells after trypsin activation (Fig. 9A panels e and g and Fig. 9B). Interestingly, 8% of nuclei were found to be in syncytia in the condition where HeLa HcRed Spike cells were co-cultured with GFP-ezrin_FERM_ cells after trypsin activation (Fig. 9A panel i and Fig. 9B). This 2-fold increase of fusion, linked to expression of GFP-ezrin_FERM_ in target cells, was consistently found in four independent experiments. This result shows that target cells expressing the FERM domain of ezrin are more susceptible to S-dependent cell-cell fusion and further indicates that ezrin plays a restrictive role during SARS-CoV S-dependent fusion process.

## Discussion

Our data demonstrate for the first time an interaction between the membrane tethering protein ezrin and the endodomain of the SARS-CoV S envelope glycoprotein and describe a novel mechanism involving ezrin as a restraining factor of SARS-CoV entry. We show that ezrin, by binding to S endodomain, limits S-dependent early events of infection, most likely by affecting efficacy of fusion. Our data point towards a novel role of ezrin as a regulator of early events of infection of susceptible cells by SARS-CoV.

### SARS-CoV S endodomain interaction with ezri

Ezrin was identified for its interaction with the SARS-CoV Spike endodomain by yeast two-hybrid screening (Fig. 1A). Analysis of sequences of prey hits has shown that the F1 lobe of the N-terminal FERM domain of ezrin mediates interaction with S endodomain (Fig. 1C–D). FERM lobes of a number of FERM-domain containing proteins have been described as mediating specific interactions with proteins or phospholipids at the plasma membrane. For example, a region located between the F1 and F3 lobes is the site where inositol-(1,4,5)-triphosphate (IP3) interacts with the FERM domain of radixin [40]. Moreover, crystallographic data have shown that sites within the F3 lobe of radixin are the binding regions of CD44 cytoplasmic region [41] and ICAM-2 [42]. In addition, moesin F3 lobe was found to interact with the EBP50 scaffolding protein [43].

Interestingly, radixin, which shares 76% amino-acid sequence identity with ezrin [24], was also identified in the yeast two-hybrid screening for its interaction with S endodomain, albeit with a lower Predicted Biological Score (B), and only three independent clones were found. The functional experiments presented here focused on ezrin exclusively; however, the fact that radixin was found in the screen suggests that other ERMs could be implicated in the life cycle of SARS-CoV. Furthermore, it was shown recently that ezrin, radixin, and moesin play differential roles in the life cycle of HIV-1, with some reports demonstrating that ERMs may negatively regulate post-entry events by affecting stable microtubules, and others that show ezrin and moesin as important for entry events of the virus [29]–[32]. Experiments on radixin and moesin should be performed to further investigate whether other ERMs have a role in the SARS-CoV life cycle.

### Confirmation of SARS-CoV S endodomain - ezrin interaction and characterization of interaction determinant

GST-pull down assays (Fig. 2) showed that the S endodomain of SARS-CoV could pull down ezrin from HeLa and Vero E6 lysates, providing evidence of specific interaction between both partners. Attempts of coimmunoprecipitation using full length SARS-CoV S and ezrin have not been successful. Difficulty to coimmunoprecipitate ERM proteins in our and others' studies could be due to the low proportion of activated proteins in cells, which, moreover, interact with actin and are therefore insoluble. GST-pull down assays using a series of GST-S endodomain mutants demonstrated that residues in the last 8 amino-acids as well as cysteines (particularly the juxtamembrane-most first cluster C1) were important determinants for binding with ezrin (Fig. 3). The positively charged amino-acids found in the very last 8 amino-acids contain a KxHxx dibasic motif that was shown to function as an ER retrieval signal [44], [45]. The positively charged amino-acids were of particular interest in our study as ERM proteins have been shown to interact with the C-terminal tails of transmembrane protein that contain juxtamembrane stretches of positively charged amino-acids [46]. The finding that the C1 cluster of S endodomain is also involved in ezrin binding *in vitro* is intriguing. Cysteine residues are highly conserved among coronaviral Spike proteins. SARS-CoV S cysteines are known to be palmitoylated and important for S association with detergent-insoluble cellular fractions and S-dependent cell-cell fusion [14], [16]. It could be argued that the GST-S_endo_ proteins, which have been purified from bacteria in our study, may not reflect the palmitoylation state of S. Whether a small fraction of S C1 clusters is not palmitoylated and free to interact with ezrin *in vivo* would need further investigation.

### Ezrin is present at the site of virus fusion

Analysis of GFP-Vpr SARSpp entry in Vero E6 cells by TIRF microscopy has revealed that after binding and endocytosis, RFP-ezrin could be associated around some of the internalized particles, perhaps surrounding the endosomal membrane (Fig. 4). Although recruitment of ezrin around endosomal membranes may not be specific to SARS particles' internalization, our result suggests that ezrin is present at the SARS-CoV fusion site. It is therefore plausible that following initiation of the pore opening, ezrin molecules come into contact with some newly accessible S endodomains. Topologically, an interaction between ezrin and S endodomains requires that the fusion pore be open. Knowing the function of ezrin in regulation of membrane fluidity, one could expect that ezrin modulates the efficacy of fusion at a step following the opening of the pore.

### Reduction of cellular levels of ezrin enhances S-mediated entry

We first studied the role of ezrin in S-mediated entry of SARSpp. Reduction of cellular levels of ezrin by gene silencing induced a marked increase in susceptibility to SARSpp by more than 12-fold (Fig. 5). Our results also show that silencing ezrin expression enhances, to a lower extent, entry of pseudotyped particles with no envelope or VSV G. A slight ∼2-fold increase in entry levels for HIV-gp-pseudotyped particles has also been observed (Supporting Fig. S1). No significant increase has been detected for influenza virus HA-pseudotyped particles. These enhancements have consistently been found to be lower than the one found for SARS-CoV S-mediated entry. The moderate enhancement effect of ezrin depletion by siRNA treatment on VSV G and HIV-gp pseudotyped particles suggests that ezrin may also have a general restricting effect on viral entry. Ezrin is a scaffolding protein that links actin cytoskeleton and cholesterol-rich lipid domains at the plasma membrane. It contributes to membrane structure and organization. First, ezrin provides rigidity to specific cortical areas of the cell by linking membranes to cortical actin. For instance, ezrin is important for extensions of lamellipodia and filopodia [47], [48]. Second, ezrin enables the organization of membrane microdomains by controlling fluidity of membrane components. This process is extremely important in the regulation of signalization by trans-membrane receptors and signaling molecules [49]–[51]. Moreover, ezrin enables the compartmentalization of signaling molecules into microclusters and prevents their free movement to restrict signaling. This has been well described for the B and T cell receptors [50], [52]. Therefore, it is possible that the membrane rigidity induced by ezrin limits viral entry in general, with a more important effect on SARS-CoV S-mediated entry, due to the specific interaction with S.

Of note, ezrin was also identified as a strong enhancer of SARS-CoV S-mediated entry in an independent functional siRNA library screen performed in the laboratory (Dr. Dongjiang Tang, Dr. Peigang Wang, BN, unpublished data). In this study, a library targeting 122 cellular genes implicated in cellular trafficking was used to characterize cell factors that regulate SARSpp entry. Remarkably, ezrin siRNAs had the second-most enhancing effect.

### Mutation of the S protein ezrin-binding domain is associated with an increase of SARSpp transduction level

The deletion of the last 8 amino acids of the S protein, which was shown biochemically to decrease interaction with ezrin (Fig. 3), allowed pseudotyped particles to achieve higher transducing levels than wt S SARSpp (Fig. 6). This finding further supports the notion that ezrin's interaction with S negatively modulates entry, although we may not exclude the possibility that higher S incorporation observed in the case of SΔ8 SARSpp also contributes in increasing viral entry. Effects of truncations of S endodomain have been analyzed in several independent studies. Petit et al. have shown that a truncated S lacking the 8 C-terminal amino-acids, named T1247 in their study, was less efficient in mediating S-driven cell-cell fusion than wt S (34.01% of wt S) [17]. This was explained by an altered surface expression of S T1247 (79.15% of wt S). In another study by Giroglou et al., although low cell surface expression of SΔ8 was also documented in 293T cells, SΔ8 SARS-MLV pseudotyped particles could be produced and could transduce Vero cells ∼10-fold more than wt S SARS-MLVpp [19]. In addition, deletion of the last C-terminal 17 amino acids increased S-mediated cell-cell fusion [17] and SΔ19 mutants were shown to be efficiently expressed at the cell surface of 293T pseudotyped particles producer cells and induced a 100-fold increase in transduction of Vero cells [19]. A study by Moore et al. has shown that a S protein variant with a deletion of the last C-terminal 27 amino-acids and addition of the eight most membrane-proximal residues of the HIV-1 envelope glycoprotein cytoplasmic domain (S-H2 in this study) induces better cell surface expression, incorporation into simian immunodeficiency virus (SIV) pseudotyped particles and capacity to mediate transduction by SIVpp than wt S [18]. The authors concluded that the greater efficiency of transduction of S-H2 pseudotyped particles compared to wt S SARS-SIVpp was likely due to preferential incorporation of S-H2. Further truncations altering cysteine residues have resulted in decreased transduction levels [18], [19] and deficiencies in cell-cell fusion [14]. Interestingly, in our study we found that SΔ8 C1 SARSpp could transduce slightly more potently Vero E6 cells than wt S SARSpp (Fig. 6). We have shown that SΔ8 C1 can no longer bind ezrin *in vitro* (Fig. 3). SΔ8 C1 was better incorporated into HIVpp than wt S (3.9-fold difference). The higher incorporation level could be partly responsible for the enhancement of transduction. However, the contribution of the absence of ezrin binding cannot be excluded in the observed enhancement of SΔ8 C1 SARSpp entry. Our work indicates that, in addition to the levels of S incorporation into pseudotyped particles and S fusogenicity, binding to ezrin may also contribute to modulate the efficiency of S-mediated entry.

### Expression of the dominant negative FERM domain of ezrin increases host cell susceptibility to infection by SARS-CoV and SARSpp and S-mediated cell-cell fusion

Expression of the dominant negative N-terminal FERM domain of ezrin in Vero E6 cells was used to investigate the consequences of ezrin function disruption on the host cell susceptibility to SARS-CoV infection and entry of SARSpp. Replication levels and rates of infection were similar for Vero E6 and Vero E6 cells expressing GFP-ezrin_wt_ (Fig. 7B–C). When the FERM domain of ezrin was expressed, cell susceptibility to infection was enhanced with either SARS-CoV or SARSpp (Fig. 7 and 8), further indicating that ezrin's restricting role on SARS-CoV infection takes place at the entry step. We speculate that ezrin acts at the fusion stage, as cells expressing GFP-ezrin_FERM_ were twice more susceptible to fuse with S-expressing HeLa cells than GFP or GFP-ezrin_wt_ Vero E6 cells (Fig. 9). Interestingly, in these experiments expression of GFP-ezrin_wt_ did not inhibit infection or fusion. As ezrin is naturally abundantly expressed in the cells used in this study, moderate expression of GFP-ezrin_wt_ has no effect simply because it does not change massively the overall cellular content of ezrin. In other experiments, we have observed that significant overexpression of GFP-ezrin_wt_ was able to enhance SARSpp transduction (data not shown). We speculate that massive overexpression of GFP-ezrin_wt_ has consequences on ezrin functions in the cell that can ultimately impact SARSpp entry. The use the dominant negative FERM domain or of siRNA appears to be more relevant to study the role of ezrin.

The fact that the replication level measured by qRT-PCR was slightly lower in the clone expressing the FERM domain of ezrin than control cells at 24 hours post-infection (Fig. 7B) suggests that although ezrin restricts the rate of infection at early time points, its function may be important for later stages. Alternatively, cell fitness may be affected in FERM expressing cells, affecting virus replication rate. We have investigated the potential involvement of ezrin on viral release using the SARS S, M, N and E-containing virus-like (VLP) particle system developed in the laboratory [53]. While we could not detect ezrin at sites of VLP budding, we have found presence of ezrin in preparations of purified VLPs. However, this incorporation was S-independent and expression of ezrin_FERM_ had no impact on release of VLPs (data not shown).

Recent studies on influenza HA and baculovirus gp64 viral glycoprotein-induced fusion have revealed that while the initial pore formation step is mainly controlled by the fusion protein, the subsequent steps of widening of the pore is a complex, multi-step mechanism involving cell metabolism and components, in particular membrane-curvature generating proteins [54], [55]. Interestingly, it was shown that the actin cytoskeleton restricts the opening of the fusion pore, and disrupting the actin network enhances fusion pore widening [55]. In light of this, it would be interesting to study ezrin's role in the fusion pore expansion induced by SARS-CoV S, considering the role of ezrin as a key actin-membrane linker and its interaction with SARS-CoV S endodomain after the fusion pore opening.

The present study has put to light an interaction between the SARS-CoV S endodomain and the plasma membrane-actin linker ezrin. Although direct evidence of the interaction occurring during early stages of SARS-CoV infection has yet to be demonstrated, our functional analysis reveals that ezrin has a limiting effect on SARS-CoV entry. Owing to topological constraints, we can put forward that ezrin binding to SARS-CoV S endodomain would occur during the fusion process, after the opening of the fusion pore. The findings that disruption of ezrin function enhances SARS-CoV S-dependent entry and S-mediated cell-cell fusion, would point towards a model in which ezrin scaffolds interactions with S endodomains, impose a limitation on membrane fluidity and act as a physical constraint restraining completion of fusion. The data shown here provides the basis for the establishment of a new mechanism of regulation by ezrin in the entry process of SARS-CoV.

## Materials and Methods

### Cell

The African green monkey kidney Vero E6, human embryonic kidney (HEK) 293T, human cervix carcinoma HeLa cell lines (ATCC, Manassas, VA, USA), and HeLa-F5, which stably express ACE2 [10], were used in this study.

### Antibodies

Rabbit polyclonal serum against the endodomain of SARS-CoV S was from Proscience (Poway, CA, USA). Mouse polyclonal serum against the SARS-CoV S was previously described [56]. Polyclonal rabbit anti-ezrin was a generous gift from Dr. Monique Arpin (Institut Curie, Paris, France) [57]. Goat anti-beta-actin antibody was purchased from Santa Cruz Biotechnologies (Santa Cruz, CA, USA). Anti-p24 antibodies were from Abcam (Hong Kong SAR, China). The secondary antibodies used were TRITC-conjugated goat anti-mouse IgG (Zymed, Carlsbad, CA, USA) and Horse Radish Peroxidase (HRP)-conjugated goat anti-rabbit and rabbit anti-goat IgG (Invitrogen, Carlsbad, CA, USA).

### Plasmid constructs and primers

The pcDNA-optS plasmid was generated from the pcDNA-optS-FLAG [58] construct. The pGEX-GST-S_endo_ plasmid is derived from the pGEX-4T1 plasmid (Pharmacia Biotech, Uppsala, Sweden) with the S endodomain cDNA inserted in frame downstream the Glutathione S-Transferase (GST) cDNA. The pGEX-4T1 based plasmids encoding GST-fused S endodomain with truncation of the last 8 or 19 amino-acids (Δ8 and Δ19), cysteine to alanine substitutions (C1, C2, C3, C4 and C1-4), both C-term truncations and cysteine to alanine substitutions (Δ8 C1-4 and Δ19 C1-4), as well as a sequence corresponding to the last 19 residues of S endodomain (ΔC), and S endodomain T1220A and K1227A point mutations, were constructed. The pGEX-GST-FERM encodes the FERM N-terminal domain of ezrin (residues 1 through 309) fused to the GST and was a gift from Dr. Monique Arpin.

For lentiviral pseudotyped particles (pp) production, the pNL4.3.Luc R^+^ E^−^ lentiviral vector, a gift from Dr. Pierre Charneau (Institut Pasteur, Paris), was used [59]. The pCI-VSVG plasmid encodes the sequence for vesicular stomatitis virus (VSV) G glycoprotein cloned in the pUC19 vector and was kindly provided by Dr. Garry Nolan (Stanford University, USA). pEGFP-Vpr, a pEGFP-C1 derived plasmid enabling fusion of HIV Vpr to GFP [60], was used to produce fluorescent GFP-Vpr SARSpp. The pcDNA-optSΔ8 and pCDNA-optSΔ8-C1 plasmids, which contain full-length optimized SARS-CoV S genes with Δ8 or Δ8 with C1 cysteine to alanine substitutions at the C-terminus respectively were synthesized and cloned by GeneCust (Dudelange, Luxembourg).

For establishment of stable cell lines, pCMV-dR8.91 lentiviral packaging plasmid [61], pcHMWS-HcRed1, pcHMWS-eGFP-IRES-puromycin and pcHMWS-eGFP-IRES-hygromycin transfer plasmids, which derive from the original pHR transfer plasmid [62], were used. pcHMWS-eGFP-ezrin_wt_-IRES-hygromycin and pcHMWS-eGFP-ezrin_FERM_-IRES-hygromycin were generated from sequences derived from pCB6-ezrin-VSV and pCB6-ezrinNter-VSV plasmids [26]. The pcHMWS-RFP-ezrin_wt_-IRES-hygromycin vector was generated similarly using a modified vector backbone in which the eGFP gene was replaced by RFP. pcHMWS-HcRed-IRES-hygromycin and pcHMWS-optS-IRES-puromycin plasmids were generated from HcRed and SARS-CoV Spike sequences derived from pcHMWS-HcRed1 and pcDNA-optS-FLAG plasmids.

### Viruses and pseudotyped lentiviral particles

SARS-CoV strain HK39849 was propagated and titrated on FRhK-4 cells as previously described [63], in Biosafety Level (BSL) 3 laboratory. Lentiviral particles pseudotyped with the SARS-CoV S (SARSpp), SARS-CoV S with Δ8 C-terminus deletion (SΔ8 SARSpp), SARS-CoV S with Δ8 C-terminus deletion and C1 cluster cysteine to alanine substitutions (SΔ8 C1 SARSpp), the VSV G (VSVGpp) envelope glycoproteins, or no envelope glycoprotein (Δenvpp), were generated as previously described [56], [64] in HEK-293T cells. VSVGpp were produced for generation of cell lines stably expressing GFP (VSVGpp GFP), HcRed (VSVGpp HcRed), ezrin_wt/FERM_ fused to GFP (VSVGpp GFP-ezrin_wt/FERM_), ezrin_wt_ fused to RFP (VSVGpp RFP-ezrin_wt_), or SARS-CoV Spike (VSVGpp SARS-CoV Spike).

### Yeast two-hybrid screening

Bait cloning and yeast two-hybrid screening were performed by Hybrigenics (Paris, France). The SARS-CoV S cDNA encoding the endodomain amino acids 1217–1255 was subcloned in the pB27 vector enabling fusion of the S endodomain with the LexA binding domain and transformed in the L40 ΔGAL4 yeast strain [65]. A human placenta random-primed cDNA library, transformed into the Y187 yeast strain and containing ten million independent fragments, was used for mating. One hundred forty seven million interactions were tested. After selection on medium lacking leucine, tryptophane, and histidine, 233 positive clones were picked. The corresponding prey fragments were amplified by PCR and sequenced at their 5′ and 3′ junctions. Sequences were then filtered and contiged [60] and compared to the latest release of the GenBank database using BLASTN [66]. A Predicted Biological Score was attributed to assess the reliability of the interaction, as described earlier [67].

### GST pull down assays

GST fusion proteins were produced and coupled to Glutathione Sepharose beads as previously described [33], with minor modifications. For competition assays, anti-SARS-CoV S endodomain rabbit serum was added.

### Generation of stable cell lines

Generation of clonal and polyclonal stable cell lines was performed according to standard procedures. Briefly, Vero E6 or HeLa cells were infected with VSVGpp enabling expression of different transgenes and selected with hygromycin B at 250 µg/mL for all cell lines except for HeLa cells expressing both HcRed and Spike, which were selected on medium containing both puromycin (5 ng/mL) and hygromycin B (250 µg/mL).

### TIRFM analysis of SARSpp entry

Vero E6 cells stably expressing RFP-ezrin were seeded onto glass bottom petri dishes (MatTek, Ashland MA, USA) and incubated overnight at 37°C 5% CO_2_ incubator. The cells were chilled on ice and concentrated SARSpp GFP-Vpr added at 33× concentration. The particles were left to bind to cells on ice for 30 minutes. The cells were washed three times with medium to remove unbound particles. To induce endocytosis of particles, warmed medium was added and the cells were kept in a 37°C humid chamber mounted on a Zeiss total internal reflection fluorescence microscope (TIRFM) for 30 minutes before recording. The fluorescent signal from both dyes was then recorded with intervals of 3 seconds. The incidence angle used was 65 degrees to enable examination of layer thicknesses of 200 nm and visualization of processes at the cell membrane. Fluorescence images were processed with MetaMorph software (Molecular Devices, Sunnyvale, CA, USA) and image sequences combined into movies (Supporting movie S1).

### Small interfering RNA treatment

Small interfering RNA (siRNA) reagents used were purchased from Dharmacon (Lafayette, CO, USA). Ezrin siRNA were designed from the human cDNA sequence of ezrin (*EZR* or *VIL2* gene, NM_003379). Ezrin siRNA pool corresponds to an equimolar mix of 4 siRNA duplexes with the following forward sequences: GCUCAAAGAUAAUGCUAUGUU, GGCAACAGCUGGAAACAGAUU, CAAGAAGGCACCUGACUUUUU, GAUCAGGUGGUAAAGACUAUU. Negative control, non-targeting (NT) siRNAs were used (pool of 4 duplexes). HeLa-F5 cells were seeded in 96-well plates, at 3×10^3^ cells per well. Twenty-four hours later, a first round of siRNA transfection was performed using Dharmafect transfection reagent and non-targeting or ezrin siRNA pools at 100 nM final concentration (Dharmacon). A second round of transfection was performed 48 hours later.

### Analysis of SARSpp entry

For siRNA experiments, forty-eight hours after the second siRNA treatment, HeLa-F5 cells were infected with lentiviral pseudotyped particles as described previously [12], [64]. Each condition was performed in quadruplicates. Luciferase activity was measured forty-eight hours post-infection using the BrightGlo luciferase substrate (Promega, Madison, WI, USA) and a MicroBeta JET luminescence counter (Perkin Elmer). Analysis of knock down of ezrin expression relative to β-actin levels was done by Western blot of pooled lysates used for luminescence measurements. For all other experiments involving pseudotyped particles, cells were infected with the respective pseudotyped particles and luciferase activity was measured as described above.

### SARS-CoV replication analysis

Cells were seeded in 24-well plates at 1.5×10^5^ cells per well and infected with SARS-CoV S strain HK39849 at an M.O.I. of 5. Three, six and twenty four hours post infection, viral gene expression was analyzed by quantitative RT-PCR as described previously [63]. Quantitative RT-PCR analysis was performed using a Light Cycler 480 (Roche). Levels of viral gene expression were normalized to cellular 18S rRNA levels.

### Immunofluorescence assay of SARS-CoV infected Vero E6 stable cell lines

Vero E6, Vero E6 GFP-ezrin_wt_ and Vero E6 GFP-ezrin_FERM_ stable cells were seeded on microscopy chamber/slides at 10^5^ cells per well (Ibidi, München, Germany). Cells were infected with SARS-CoV strain HK39849 at M.O.I. of 5. Twenty four hours after infection, cells were fixed and SARS-CoV S, actin or nuclei were labeled using specific antibodies, AlexaFluor 555-conjugated Phalloidin (Invitrogen), or 4′,6′–diamidino-2-phenylindole (DAPI), respectively and mounted using Fluoromount G (Southern Biotech), according to standard procedures. Microscopy observations and image acquisitions were performed using a Zeiss AxioObserver Z1 microscope (10× objective). For each cell line studied, 10 random fields were acquired. Counting of total number of cells (DAPI or Phalloidin staining) and SARS-CoV infected cells (S-TRITC staining) was performed by Imaris 6.3 software (Bitplane, Zurich, Switzerland), enabling calculation of percentage of SARS-CoV infected cells.

### Cell-cell fusion assay

HeLa cells stably expressing HcRed or HcRed and Spike were seeded onto glass coverslips in 24-well plates at a density of 0.75×10^5^ cells per well. After 16 hours, Vero E6 cells stably expressing GFP, GFP-ezrin_wt_ or GFP-ezrin_FERM_ were overlaid at the same density. Six hours later, cells were treated or not with 10 µg/mL of L-(tosylamido-2-phenyl) ethyl chloromethyl ketone (TPCK)-treated trypsin (Sigma) at 37°C for 15 minutes to activate S protein. After addition of FCS-containing medium to inactivate TPCK trypsin, the cells were incubated for 18 hours at 37°C 5% CO_2_, then fixed with 2% PFA, and stained with DAPI. The coverslips were mounted onto microscopy slides and analyzed using Zeiss AxioObserver Z1 microscope with 40× objective. For each condition, 10 random fields were acquired and analyzed using MetaMorph image analysis software (Molecular Devices). Total number of nuclei (DAPI staining) and number of nuclei contained in multi-nucleated cells (DAPI/HcRed/GFP positive cells) were counted, enabling calculation of percentage of nuclei involved in syncytia formation.

## Supporting Information

Movie S1 **Ezrin accumulates at sites of entry of SARS-CoV S-pseudotyped lentiviral particles.** Vero E6 cells stably expressing RFP-ezrin_wt_ were seeded onto a microscopy culture dish and were inoculated with SARSpp harboring a GFP-tagged Vpr protein (SARSpp GFP-Vpr) on ice. Unbound particles were washed with cold medium. The culture dish was then placed in a 37°C 5% CO_2_ chamber of a Total Internal Reflection Fluorescence (TIRF) microscope for 30 minutes to allow for internalization of particles. After 30 minutes, time-lapse images were acquired to follow the movements of SARSpp GFP-Vpr particles during the internalization process at and beneath the plasma membrane of Vero E6 cells expressing RFP-ezrin. Fifty frames were acquired at 3 seconds intervals. Scale bar = 40 µm.
(MOV)

Figure S1 **Entry of VSV G (VSVGpp), HIV envelope gp (HIVpp), and Influenza HA (HApp) pseudotyped particles in ezrin siRNA-treated cells.** HeLa P4 (**A**), and Huh-7 (**B**) cells were subjected to treatment with ezrin siRNAs as previously described in the manuscript's materials and methods section. The cells were then infected with VSVGpp (**A, B**), HIVpp (**A**), and HApp (**B**) lentiviral pseudotyped particles. Activity of luciferase was measured after 72 hours and results correspond to fold-change compared to entry in non targeting siRNA-treated cells (average of triplicates).
(TIFF)
