# Supplementary figures and images for: Ezrin Interacts with the SARS Coronavirus Spike Protein and Restrains Infection at the Entry Stage
Source: PLoS One. 2012 Nov 21;7(11):e49566. doi: 10.1371/journal.pone.0049566 (PMC3504146; doi:10.1371/journal.pone.0049566)

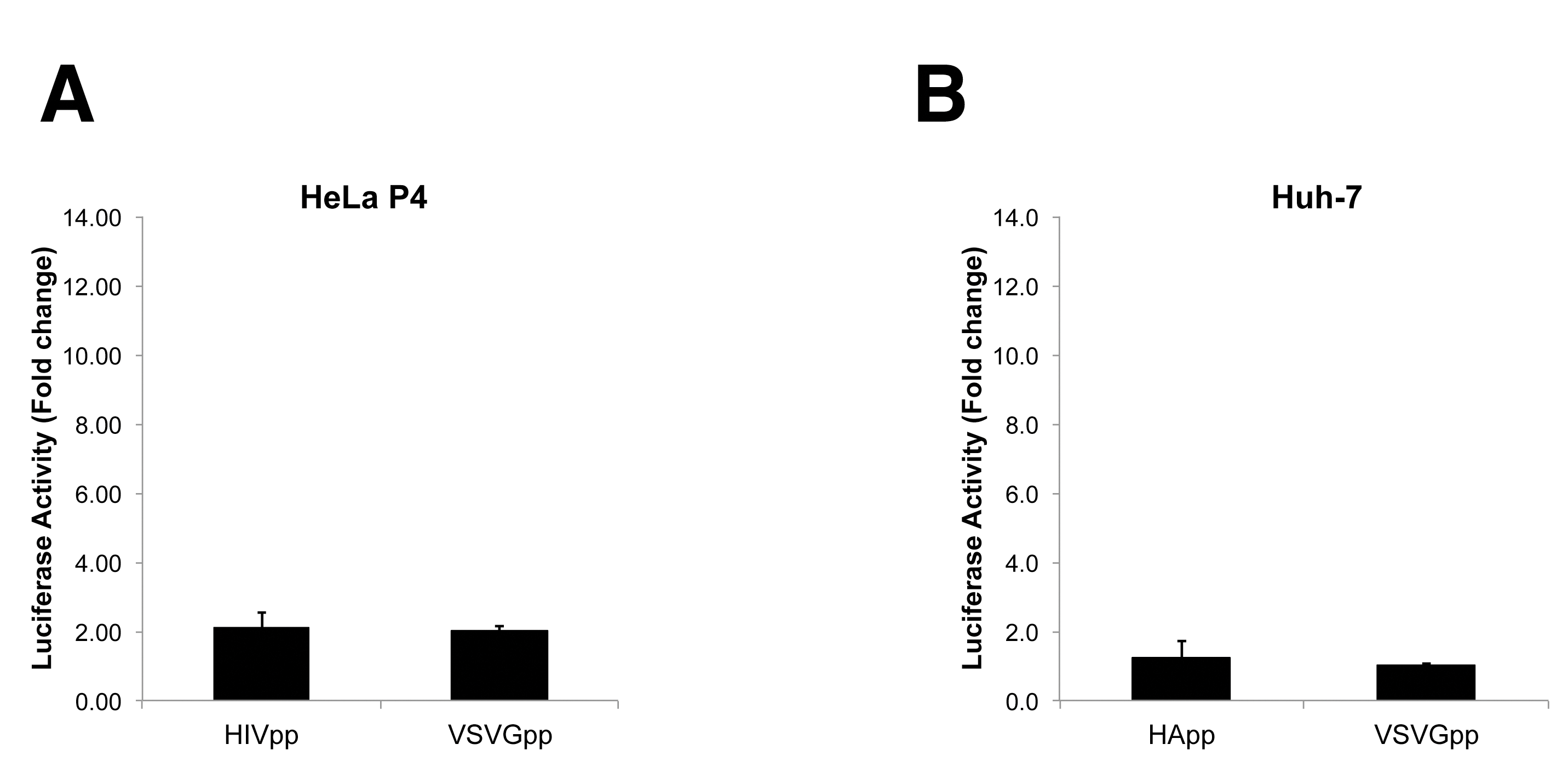

Supplement: Figure S1 — Entry of VSV G (VSVGpp), HIV envelope gp (HIVpp), and Influenza HA (HApp) pseudotyped particles in ezrin siRNA-treated cells. HeLa P4 (A), and Huh-7 (B) cells were subjected to treatment with ezrin siRNAs as previously described in the manuscript's materials and methods section. The cells were then infected with VSVGpp (A, B), HIVpp (A), and HApp (B) lentiviral pseudotyped particles. Activity of luciferase was measured after 72 hours and results correspond to fold-change compared to entry in non targeting siRNA-treated cells (average of triplicates). (TIFF) [file pone.0049566.s002.tiff]
